# Assessing patterns of authorship of low- and middle-income countries in global commercial clinical trials in oncology

**DOI:** 10.1186/s12992-025-01167-8

**Published:** 2025-11-22

**Authors:** Anil Babu Payedimarri, Samir Mouhssine, Saleh Aljadeeah, Blaise Mwizerwa Nkubito, Gianluca Gaidano, Raffaella Ravinetto

**Affiliations:** 1 Division of Hematology, Department of Translational Medicine, University of Eastern Piedmont and AOU Maggiore della Carità di Novara, Novara, Italy; 2 Department of Public Health, Institute of Tropical Medicine, Antwerp, Belgium; 3 Independant researcher, Namur, Belgium; 4 School of Public Health, University of the Western Cape, Cape Town, South Africa

**Keywords:** HICs, LMICs, UMICs, Authorship patterns, Clinical trials, Global health, Oncology, Breast, Lung, Colon, Trend, Inequalities

## Abstract

Poor authorship practices in global health research may be a sign of unequal partnerships. Previous studies have shown that authors from low- and middle-income countries (LMICs) are frequently underrepresented in publications from global research collaborations between LMICs and high-income countries (HICs). To the best of our knowledge, the patterns of authorship from LMICs in international industry-sponsored clinical research on breast, lung and colon cancer have not yet been investigated. Therefore, as a spin-off to broader research on globalization of commercial clinical trials in oncology, we conducted an analysis of authorship in the publications from completed industry-sponsored therapeutic trials in breast, lung and colon cancer (from phase I to IV) that involved LMICs. Only articles published in a peer-reviewed journal in English by March 30, 2024 were included. A total of 302 publications from 173 trials were analysed. 63% (*n* = 191) of them have at least one author from a middle-income country (MIC); 14% (*n* = 42) articles have the first author from a MIC; and 13% (*n* = 39) articles have the last author from a MIC. Conversely, 37% (*n* = 111) of articles had no author from MIC, including two trials conducted only in MICs. In conclusion, our study found an imbalance in authorship, suggestive of significant inequalities, in collaborative research in industry-sponsored clinical trials for breast, lung and colon cancer. Industry sponsors need to work towards greater equity in authorship when collaborating with researchers in (L)MICs, and oncology researchers and opinion leaders in HICs should actively advocate for greater fairness to their colleagues in (L)MICs.

**Supplementary Information:**

The online version contains supplementary material available at 10.1186/s12992-025-01167-8.

## Introduction

According to the International Agency for Research on Cancer (IARC), breast, lung, and colon cancers were the three most frequent cancers in 2022, accounting for more than 30% of new cases and about 34% of deaths each year [1]. For this reason, they are referred to as the “big killers” in oncology [2]. Consistently, they represent a major topic in cancer research globally.

The importance of building global health research capacity in low- and middle-income countries (LMICs) is increasingly recognised [3, 4]. The World Health Organization (WHO) has made recommendations for greater equity in health research, pointing to the unbalanced distribution of power, money and resources globally [5]. Although cancer is a relevant health problem in LMICs, the inequalities in access to adequate diagnosis and treatment are mirrored by inequalities in oncology research, including research governance and ownership. For instance, between 2014 and 2017, almost 30% of randomized controlled trials (RCTs) in oncology involved patients from LMICs, but only 8% of trials were led by authors from LMICs, while the remaining 92% were led by authors from high-income countries (HICs) [6, 7].

The number of clinical research collaborations between HICs and LMICs has increased over time in different medical fields and disciplines [8]. However, disparities persist in professional recognition, as shown by the publication patterns of research findings [3, 4, 9]. In global health research, fair research partnership is essential. First, the pursuit of equity and inclusion is critical to ensure that research is impactful and locally applicable [10, 11]. Second, as highlighted in the Global Code of Conduct for Equitable Research Partnerships (TRUST CODE), recognising scientific credit to the investigators from LMICs is per se a matter of integrity and fairness (“Article 4: Local researchers should be included, wherever possible, throughout the research process, including in study design, study implementation, data ownership, intellectual property and authorship of publications”) [12]. However, the unequal power dynamics in global health research have been widely documented, as well as their consequences [13–15].

A symptom of these inequalities is the unfair authorship pattern frequently observed in publications in global clinical research, including, but not limited to, in cancer research. Also in other international health research projects involving LMICs, first and last authorship often appears to be mainly allocated to HIC authors [16, 17]. Authors of research papers including LMICs and affiliated with institutions in LMICs account for a fraction of all first authors [18–23], and are underrepresented as first and last authors [3, 24]. Some studies that specifically analysed authorship practices in clinical trials have raised concerns about the agency of local partners in global health collaborations and the fairness of partnerships therein [25, 26]. Few other studies and reports investigating authorship practices in global health research also pointed at imbalances in regional representation and in the distribution by country income groups [15, 16, 27]. These findings highlight persistent inequalities in authorship practices in global health research.

Some authors have explained this phenomenon with the lack of economic resources and/or infrastructure, weak academic institutions, lower experience in manuscript drafting, language barriers, and biased judgement of LMIC authors and research topics by scientific journals [28]. The majority of governments in LMICs [and particularly in low-income countries (LICs)] lack substantial resources towards research, leading to insufficient strengthening and ownership of academic and research institutions [29]. As a result, local universities aiming to foster a research-oriented environment must collaborate with HIC institutions to obtain funding [9]. Noormahomed et al. also suggested that only a limited number of mentors possess a strong grounding in research within LMIC institutions [30]: hence, despite research methods being included in curricula, and positive trends toward capacity strengthening, a gap would persist between theory and practical applications. Another challenge confronted by researchers from some LMICs revolves around the fact that they may not be proficient in English, that is the language commonly used in medical publications [13]. This limitation places them at a disadvantage when it comes to effectively conveying their findings. Authorship of published research has a direct impact on hiring, tenure, promotion, and award decisions in academia [28]. Especially first and last authors are sought after, as these positions are associated with greater professional recognition, which in turn leads to greater funding opportunities and career advancement [31]. The inequity in authorship practices thus contributes to a vicious circle, where researchers from LMICs faces extra challenges to emerge compared to their peers in HICs. A recent study found that among 164 National Cancer Institute (NCI) funded grants involving LMICs, 51% of all publications did not include any author affiliated with an institution in an LMIC. Additionally, 78% and 83% of publications had a first or last author, respectively, affiliated with a HIC [32]. Another cross-sectional study found that Sub-Saharan African authors are underrepresented in global oncology articles [33].

All these facts contribute to explain why LMICs researchers may have to rely heavily on industry sponsors to conduct and/or to participate in the clinical trials [34], particularly in highly-specialised fields such as oncology. Although oncology commercial clinical trials, e.g. oncology trials sponsored by the pharmaceutical industry, frequently involve sites and co-authors from LMICs, the leadership roles is mainly reserved to the authors from HICs [34, 35]. As discussed above, trends in authorship have been described in other medical specialities (such as infectious diseases and some oncologic diseases) [9, 35]. To the best of our knowledge authorship distribution assessment has not been done in international industry-sponsored clinical research for breast, lung and colon cancers. Since a recent study from our group documented the globalization of breast, lung, and colon cancer interventional industry-sponsored therapeutic clinical trials [36], we have now embarked on a sub-analysis to describe the trends in the allocation of authorship in such trials. In particular, we seek to characterise allocation of authorship [by affiliation of the first, co-, and last author(s)] to investigators in LMICs across breast, lung, and colon cancer industry-sponsored clinical trials, as an indicator of fairness in research collaborations.

## Methods

In our study, globalization of commercial clinical trials is defined as clinical trials sponsored by a pharmaceutical industry and conducted as part of multinational (multiple sites across more than one country) clinical development for the regulatory approval of new medicines.

### Articles search strategy

As described elsewhere [36], we conducted a structured search in ClinicalTrials.gov, an open-access trial registry [https://clinicaltrials.gov/]. The advanced search strategy combined the following fields: *(i)* study type: interventional trials; *(ii)* condition/disease: breast cancer, lung cancer, colon cancer; *(iii)* phases: Phase I, II, III and IV; *(iv)* Type of funder: industry; *(v)* first posted: registry start date. The main data extraction occurred on June 30, 2018. For the scope of this follow-up study, we conducted a second data extraction on September 30, 2022, adding the following fields: *(vi)* study completion, *(vii)* study results-(with results, without results) and *(viii)* publications. After the second data extraction, we searched and downloaded all articles (of each trial record) available in the registry for completed trials, based on the registry field termed “Publications”. Third, we manually searched PubMed and Google Scholar using the trial identifier (NCT number, which is the identification code in ClinTrial.gov) for any other publications related to each trial. We searched for all articles published (for each trial record) up to 30 March 2024.

### Inclusion and exclusion criteria

We included all completed industry-sponsored therapeutic trials involving LMICs that had publications (from phase I to IV), and tested medicines in breast, lung, and colon cancers. For each completed trial we included only the original research papers reporting the trial results and articles reporting study design, such as the trial protocol. The search was limited to publications in English in peer-reviewed scientific journals as of 30 March 2024.

Completed trials conducted only in HICs (whether with publications or not) and completed trials involving (also) LMICs that did not have publications (yet), were excluded. Any case reports, reviews, systematic reviews and/or meta-analysis articles, papers reporting cost-effectiveness analysis, pooled analysis of multiple trials were also excluded.

### Articles selection and countries classification

After selecting the eligible publications, we looked at authorship patterns: *(i)* whether researchers from each LMIC involved in the trial were co-authors at all, and *(ii)* whether they were co-authors, first authors, or last authors.

The authorship allocation was categorized based on the “affiliation” section of the article. The country’s income status [High-income countries (HIC), Upper-middle-income countries (U-MIC), Lower-middle-income countries (L-MIC) or Low-income countries (LIC)] was extrapolated from the World Bank’s Country Income Classification status (fiscal year 2019) [37]. If an author was affiliated with both HICs and LMICs, he or she was assigned and considered as an HIC affiliation, as it has been done in previous bibliometric studies [9]. The rationale for this decision in our study is based on the theoretical resource advantages that a HIC affiliation would provide. If an author had more than one LMIC affiliation, the first affiliation listed was assumed to be the primary affiliation.

### Data extraction and analysis

Two independent evaluators (**ABP and BM)** screened all retrieved publications according to the inclusion and exclusion criteria. For each eligible article, the following variables were extracted as of March 30, 2024: Journal name, digital object identifier (DOI) number, total number of authors by country group (HIC, U-MIC, L-MIC, LIC), and LMIC author position (co-author, first author, and last author). For each article, the section on “acknowledgement” was also considered, to see if/how MIC investigators are acknowledged. The variables for articles that clearly met the inclusion criteria and did not meet the exclusion criteria were immediately recorded in a dedicated EXCEL database. Articles for which one evaluator was unsure whether they should be included were discussed by two evaluators, and disagreements were resolved by involving a third evaluator (**SM**).

The analyses proceeded in two steps. First, we described and analysed the proportion of articles from studies conducted (also) in LMICs that involved at least one researcher with affiliation in a LMIC as a co-author (LMIC author). Second, we analysed and described the proportion of articles from studies conducted (also) in LMICs that had a LMIC first author; the proportion of articles with at least a MIC middle author; and the proportion of articles with MIC last author. The statistical analyses were conducted in SPSS v.25.

## Results

### Trials and publications selection

**Fig. 1:**
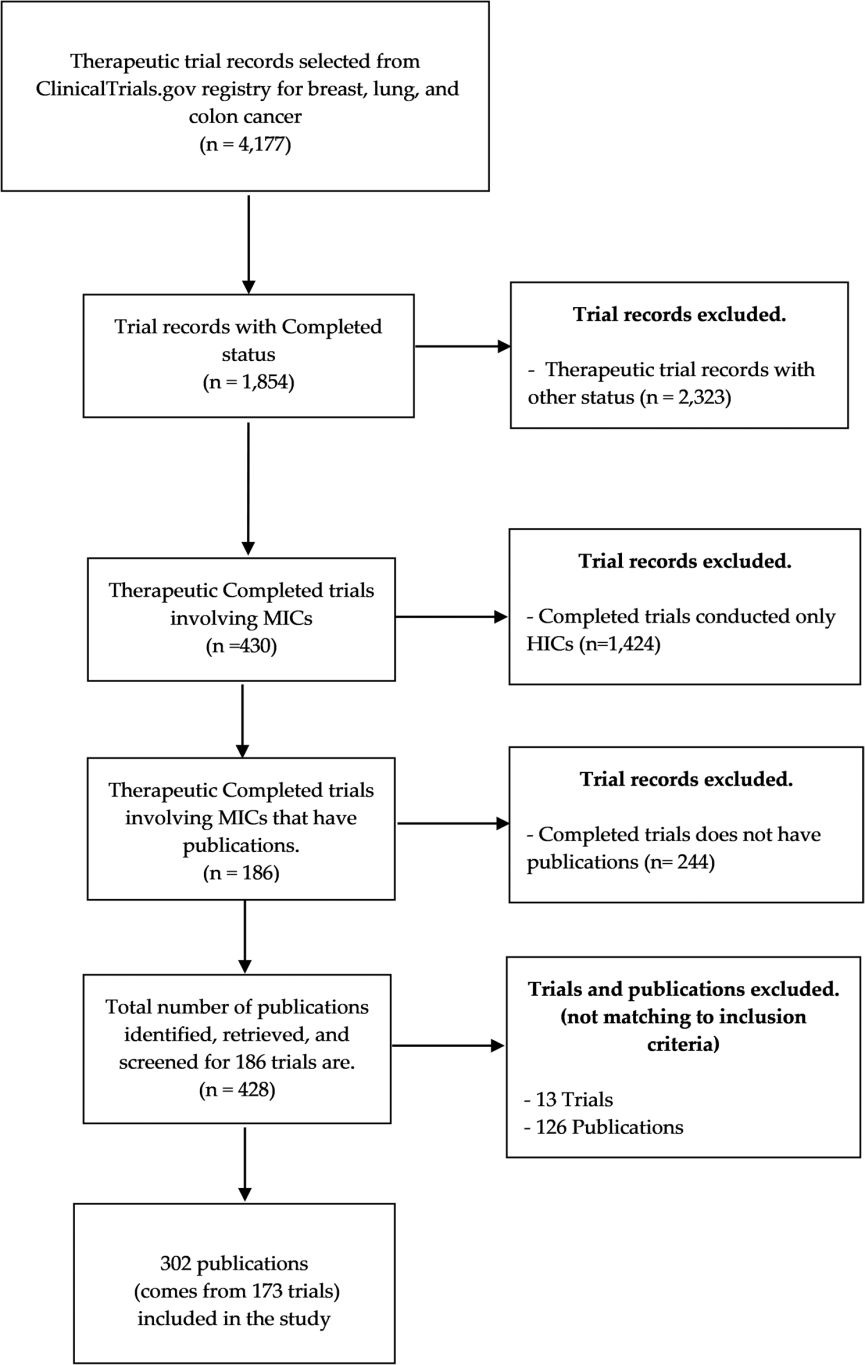
Flow chart for the selection of trial records and publications

We selected 4,177 records of therapeutic trials from the database. After removing trial records with a different status (not yet completed at the time of data extraction), 1,857 completed trials remained. After removing the 1,424 trials conducted only in HICs, 430 trials involving sites in LMICs were retained; all of them involved MICs only (and not any LICs). Out of these 430 trials, 186 trials had publications that were eligible for further screening. Out of these 186 trials, we identified and retrieved 428 publications. After full-text screening, we excluded 13 trials (because that did not have publications) and 126 publications that did not fulfil our inclusion criteria. For example, the excluded publications (in which trial identifiers were cited within the article) were published as reviews, systematic reviews and/or meta-analysis, papers reported on cost-effectiveness analysis, and pooled analysis of multiple trials. Finally, we included and analysed 302 publications (from 173 trials) (Fig. 1). The complete list with the number of trials and their clinical sites in which MICs were involved are reported in Supplementary Figure S1 (A-B). Briefly, among the L-MICs, most trials were conducted in India (*n* = 37), followed by Ukraine (*n* = 29), the Philippines (*n* = 16) and Egypt (*n* = 13). Among the U-MICs, most trials were conducted in the Russian Federation (*n* = 81), followed by Brazil (*n* = 76), China (*n* = 59) and Mexico (*n* = 53).

**Fig. 2:**
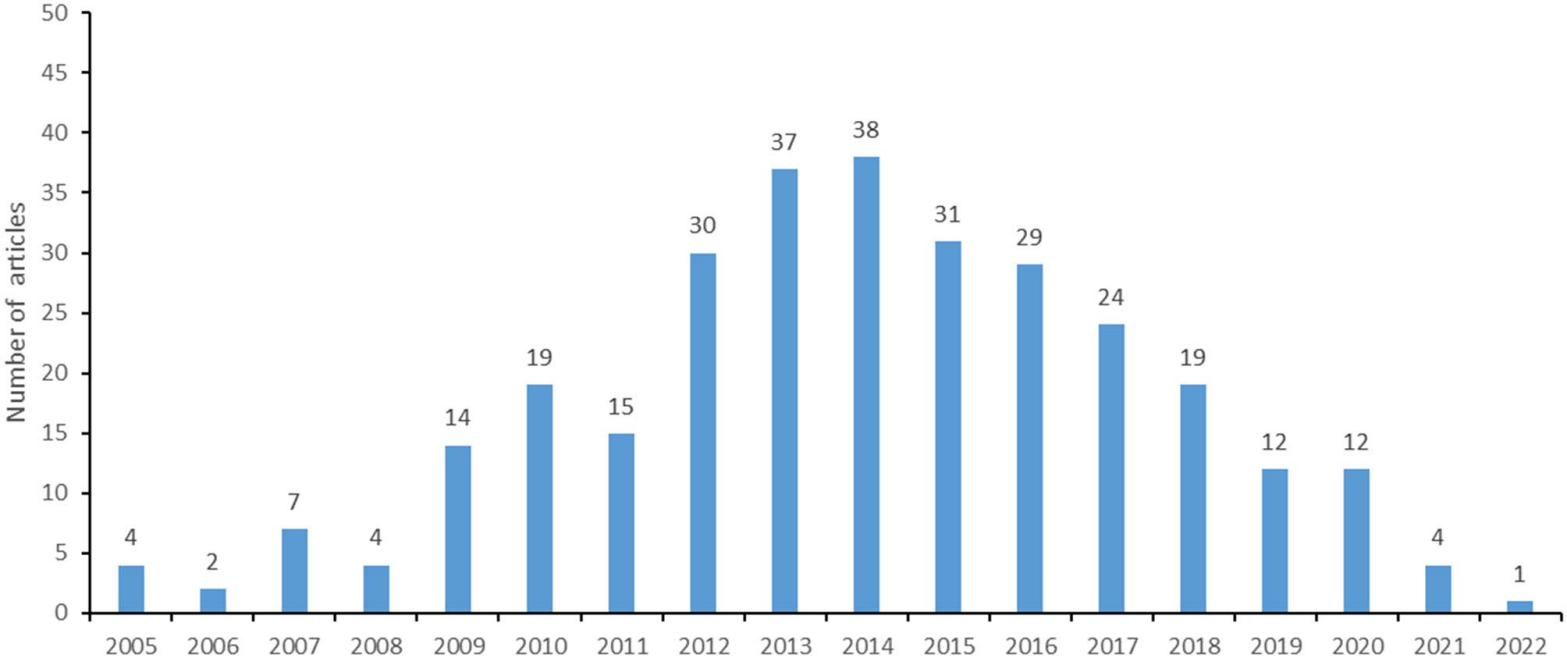
Number of articles published by year

As shown in Fig. 2, the articles included in our study were published between 2005 and 2022. The year with the highest number of publications (*n* = 38) was 2014, followed by 37 articles in 2013 and 31 articles in 2015. A ‘positive’ trend in publication numbers over the period 2006–2014, in line with the general phenomenon of globalization of clinical trials, was followed by a clear and constant decrease (2014–2021).

### Authorships trends in global oncology research

Table 1 shows the percentage of articles by authorship position. There were no studies conducted (also or only) in low-income countries. Of 302 publications, 275 articles from 153 trials involving both HICs and MICs were published in collaboration with authors from HICs and MICs. No authorship position was assigned to MIC authors in 40% (*n* = 109) of the articles. Of 20 trials conducted exclusively in MICs, 27 articles were published. Of these, 78% (*n* = 21) had a first, middle and/or last MIC author. In 7% (*n* = 2) of the articles there were no MIC authors, although the trial was conducted in MIC(s) only.

**Table 1:** Proportion of articles segregated by authorship position: HICs + mics trials vs. Only mics trials

MIC Authorship position	HICs + MICs 153 trials (*n* = 275 articles)	%	Only MICs 20 trials (*n* = 27 articles)	%	*p*-value
First author only	4	1	0	0	1
Middle author only	136	49	2	7	< 0.0001
Last author only	2	1	0	0	1
First, middle and last authors	7	3	21	78	< 0.0001
First and middle authors only	8	3	2	7	0.22
Middle and last authors only	9	3	0	0	1
No MIC authorship	109	40	2	7	0.0006
**Total**	**275**		**27**		

MICs: Middle-income countries; HICs: High-income countries

Among the 302 publications included in our analysis, 63% (*n* = 191) (Fig. 3A) of the articles have at least an author from a MIC, out of which 42 articles included the first author and 39 articles included the last author. Of the 191 articles, 76% (*n* = 146) had co-authors with affiliation in upper-MIC (UMIC) only, followed by 16% (*n* = 31) with co-authors with affiliation in both U-MIC and low-MIC (L-MIC), and 7% (*n* = 14) had co-authors with affiliation in L-MIC authors only (Fig. 3B).

Conversely, 37% (*n* = 111) of the articles have no author from MIC, including two trials conducted in MICs only (Ukraine and Turkey respectively) (Fig. 3A). In 10 of such 111 articles, MIC researchers in general (but no researcher’s name) were quoted in the “Acknowledgments” section, and in five articles the name of the study group was mentioned in the “Affiliation” section of the article.

**Fig. 3A:**
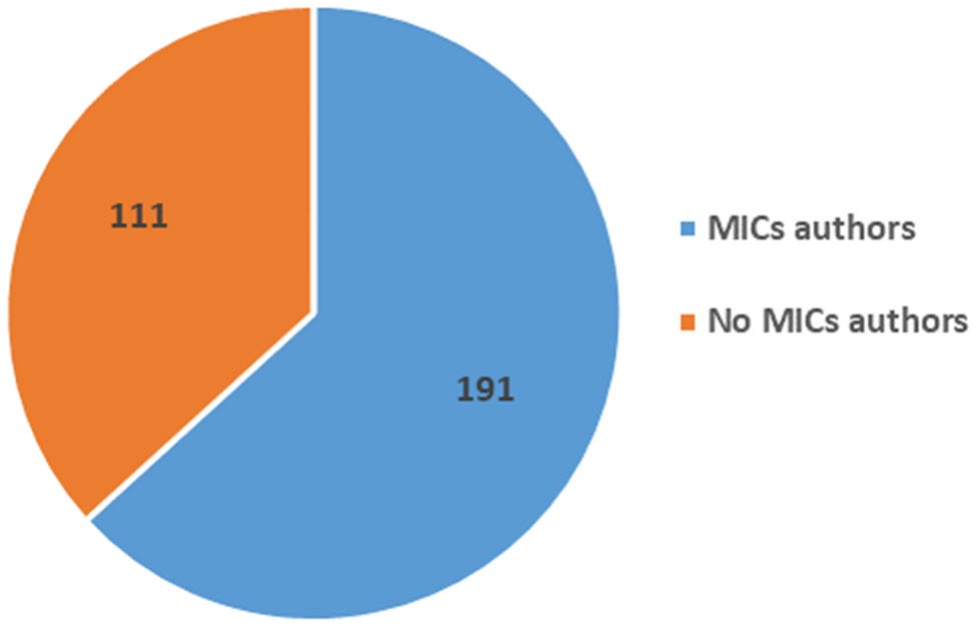
Number of articles, authors with affiliations in MICs (*n* = 302). MICs: Middle-income countries; U-MICs: Upper-middle-income countries; L-MICs: Lower-middle-income countries

**Fig. 3B:**
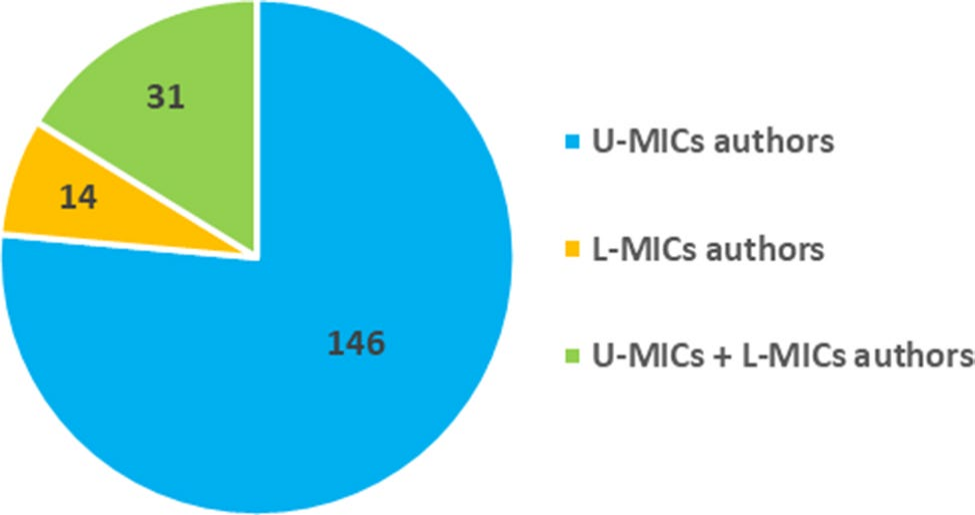
Number of articles, authors with affiliation in U-MICs vs. L-MICs authors (*n* = 191). U-MICs: Upper-middle-income countries; L-MICs: Lower-middle-income countries

#### First author

Figure 4A illustrates the temporal trend in the number of articles (*n* = 42) with a MIC researcher as first author. Noteworthy, 23 out of these trials were conducted in MICs only. The highest number of articles with a MIC researcher as first author was 7 (7/42, 17%) in 2013 and 2014, while it was 6 (6/42, 14%) in 2015, followed by 5 (5/42, 12%) in 2012. Even if less clear than in Fig. 2, also in this case there was a ‘positive’ trend (2009–2014), followed by a steady decrease (2014–2021).

**Fig. 4A:**
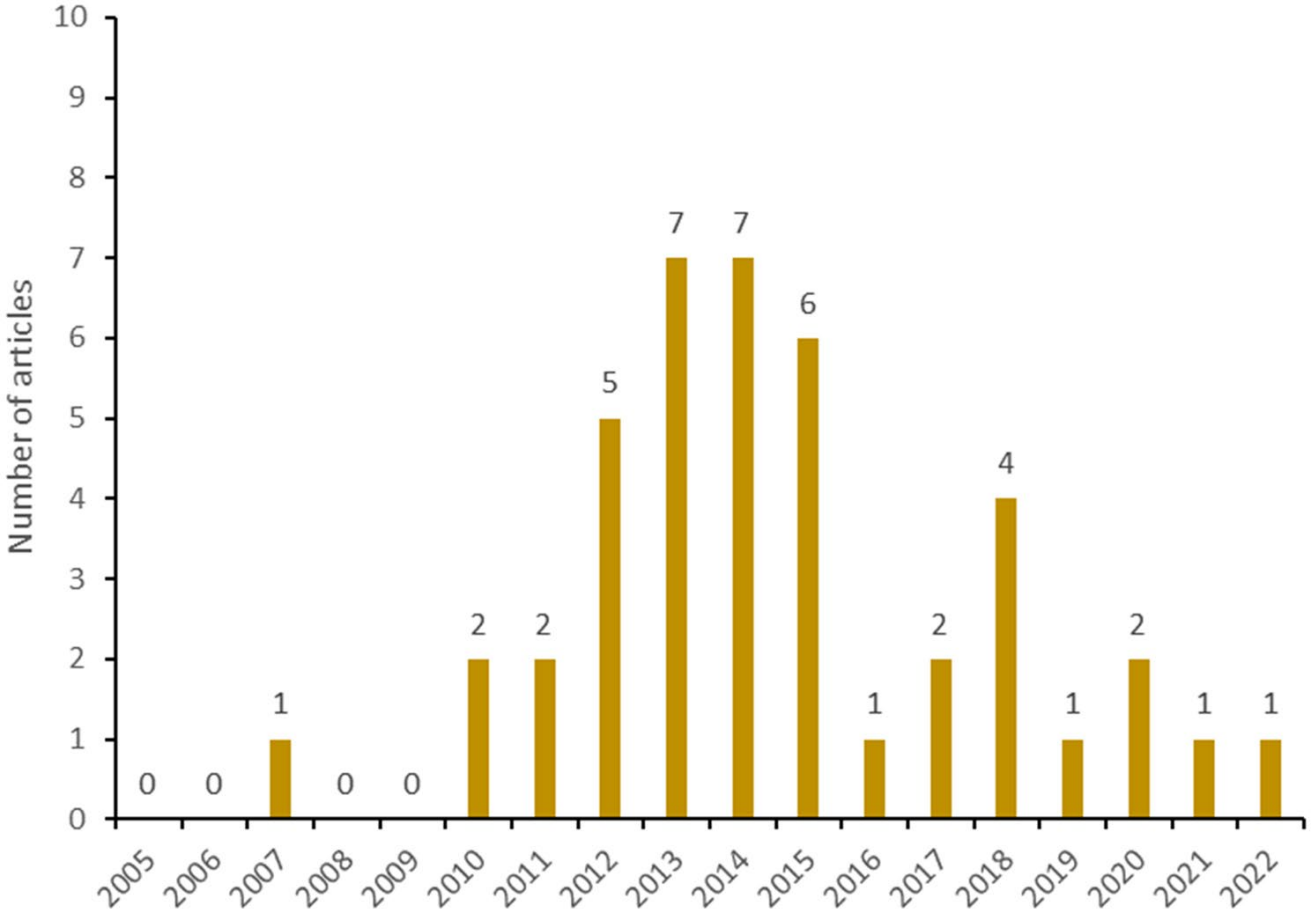
Number of articles (*n* = 42) with first author from an MIC: from 2005–2022

#### Middle author

Figure 4B illustrates the number of articles (*n* = 185) with a MIC researcher as a middle author between 2005 and 2022. The highest number of articles with a MIC researcher as a middle author was 24 (24/185, 13%) in 2013, while it was 23 (23/185, 12%) in 2014, followed by 18 (18/185, 10%) in 2015. Again, a ‘positive’ trend (2006–2013) is followed by a clear and constant decrease (2014–2021).

**Fig. 4B:**
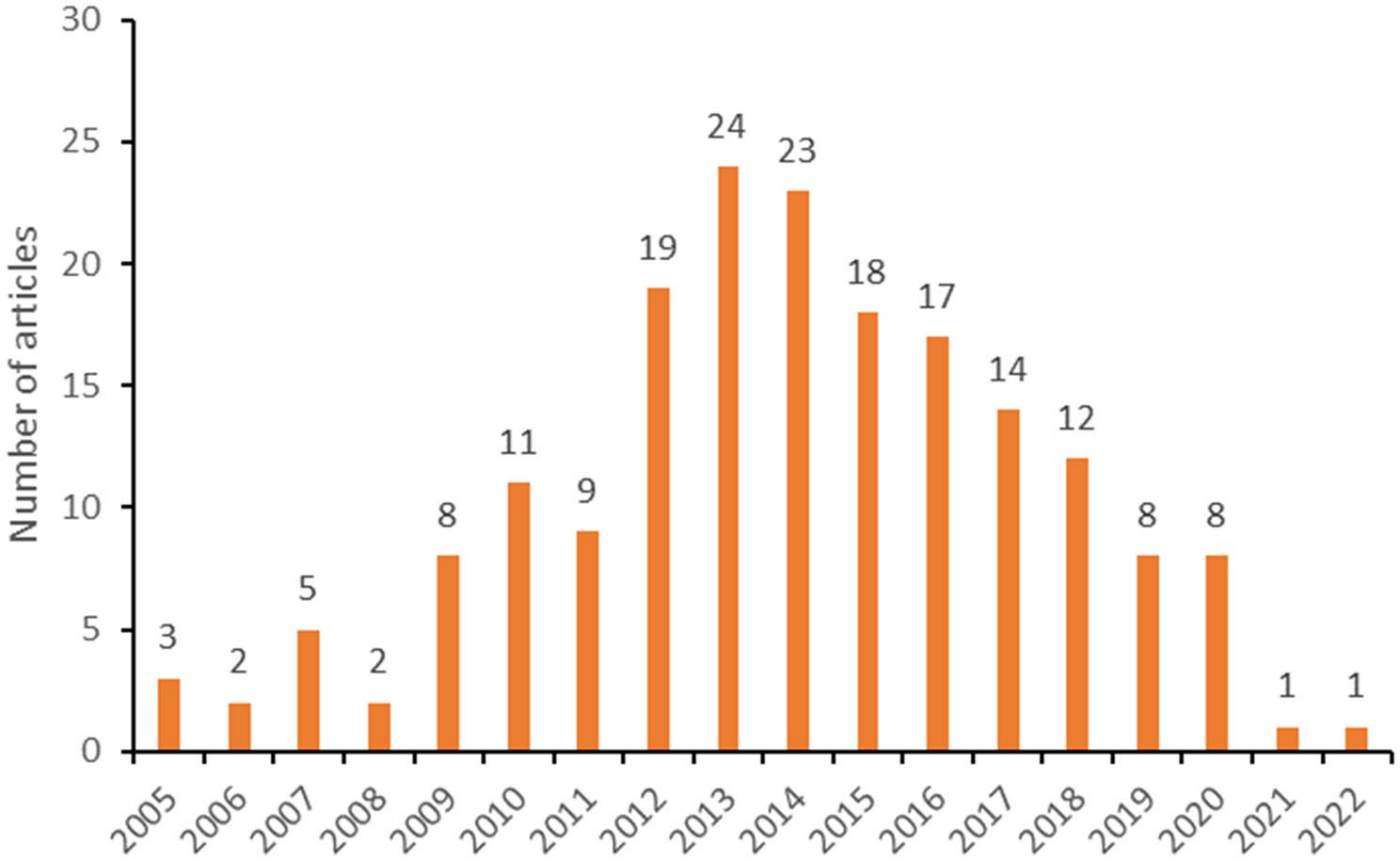
Number of articles (*n* = 185) with middle author from an MIC: from 2005–2022

#### Last author

Figure 4C illustrates the number of articles (*n* = 39) with a MIC researcher as last author between 2005 and 2022. None of these trials were conducted in MICs only. The highest number of articles with a MIC researcher as last author was 7 (7/39, 18%) in 2015, 5 (5/39, 13%) in 2013 and followed by 4 (4/39, 10%) in 2014. Temporal trends are less evident here, but one can observe highest levels around 2013–2015, followed by a steady decline in the following years.

**Fig. 4C:**
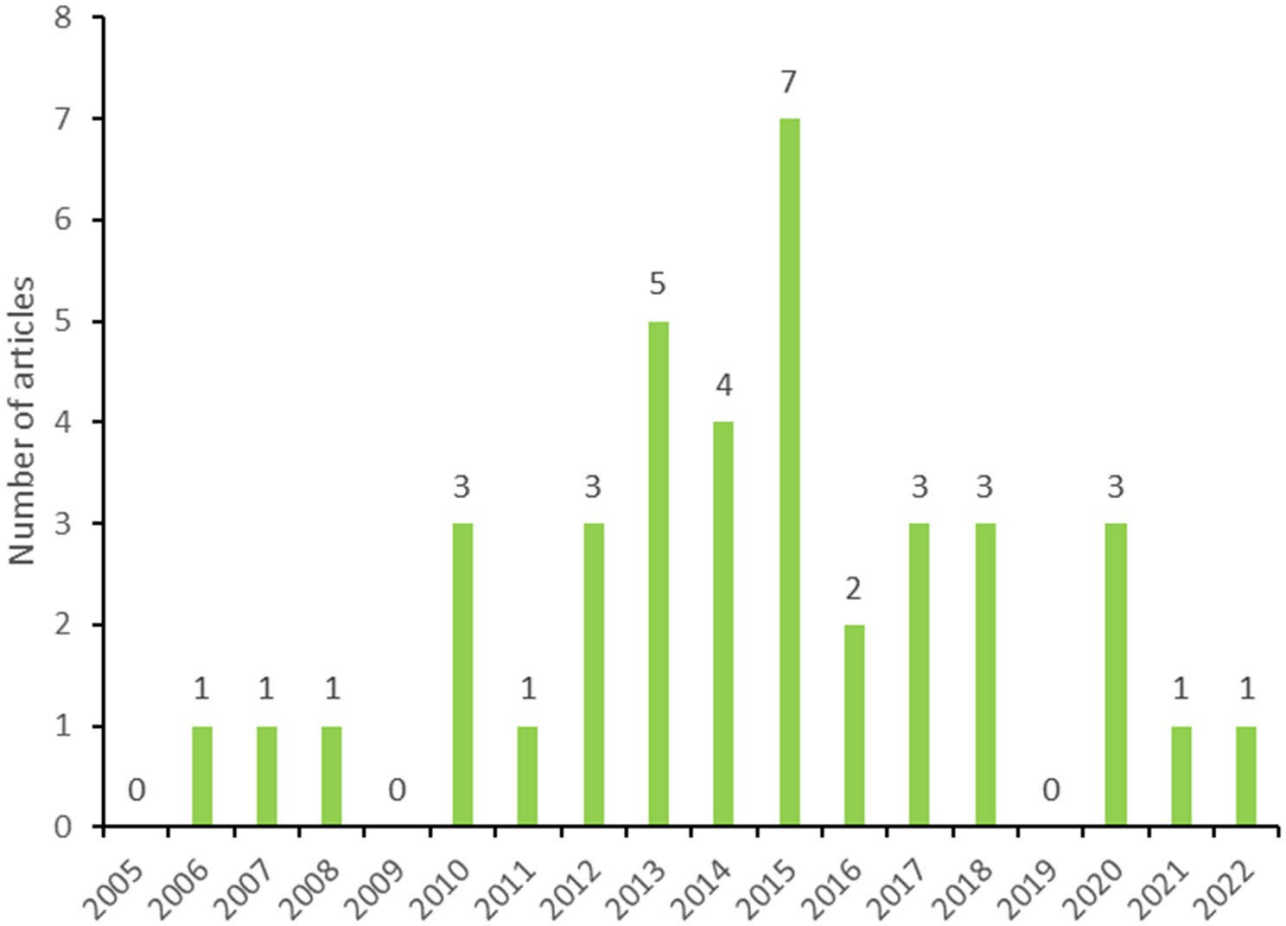
Number of articles (*n* = 39) with Last author from an MIC: from 2005–2022

## Discussion

In our study, we have explored the allocation of authorship of international industry-sponsored trials on breast, lung and colon cancer, including at least a site in LMICs. We have chosen to look at the issue of research representation through the lens of authorship, as this serves as a proxy for the research fair opportunities available as well as for the recognition of the leadership role of the authors involved in the research– in line with other authors [9]. We have also chosen to focus particularly on co-authorship and then first and last authorship, as these are considered leading positions for published research; previous work on this topic has also used this accepted convention [3, 24]. In doing so, several findings have emerged, that either confirm previous findings of other authors, or point to new elements. Noteworthy, these findings only concern clinical trials conducted in MIC, as we did not retrieve any publications concerning research involving LICs, where the power unbalance of local researchers might be greater.

First, our analysis revealed that MIC authors had no co-authorship at all in (111 articles out of 302), including two studies conducted in MICs only. The insufficient representation and inclusion of MIC authors seems evident in our dataset of industry-sponsored clinical trials on breast, lung and colon cancer, and it is suggestive of a structural imbalance in authorship in global oncology.

Second, 57% (244/430) of completed clinical trials had not (yet) published their results in peer-reviewed journals (as of 30 March 2024). Even if for more recently-completed trials this is explained by the insufficient time to get papers peer-reviewed and published, the high percentage could also be suggestive of a possible publication bias [38, 39].

Third, in our analysis, we found that in the majority of published papers, researchers from L-MICs and U-MICs are not generally represented in the position of lead author (first or last), differently from their consortium peers in HICs. This raises various questions, e.g. whether the research findings truly reflect the priorities and needs of the local stakeholders in MICs, if the lead authorship is elsewhere [3, 40, 41].

Other possible questions are whether there is sufficient awareness of/ commitment to global health equity in these research consortia, and whether the positions of lead author are systematically determined in a collaborative way, in line with the guidelines of the Committee on Publication Ethics (COPE) and of the International Committee of Medical Journal Editors (ICJME) guidelines [31, 42, 43]. While commercial sponsors have a substantial share of power, and thus play a key-role in defining the publication and authorship practices, the researchers/principal investigators from HICs involved in international, industry-sponsored research consortia also need to critically consider how and to which extent scientific credit is recognised to peers from MICs [3, 40]. Global research collaborations should build in key-aspects of social justice, namely the avoidance of unequal power relations, the promotion of group recognition, self-development and inclusive and equitable inclusion in decision-making processes [44]. We contend that researchers and opinion leaders from HIC, including in oncology field, can and should be vocal to promote equity in global health research. Moreover, if higher representation is critical to realising global health equity, regulators and other concerned policy-makers must also encourage fair and more equitable representation [41, 45]. Research Ethics Committees (RECs) (both in the host countries and in the country of the sponsor) may also play a role by requiring equitable representation in research consortia, including authorship practices [46]. The same could apply to National Regulatory Authorities, who are responsible for in-country trial oversight, and that could promote or require rigorous measures for fair research partnership, including in data co-ownership and in authorship practices. Similarly, academic journals should pursue similar avenues to enhance fair authorship representation [47, 48], by requiring and checking rigorous and substantial adherence to ICJME and COPE guidelines, including in oncology, where knowledge and awareness seem to be only recently emerged.

Previous studies have shown that research priorities in LMICs may be better represented in investigator-initiated trials targeting cancer types that are more prevalent in these countries [49, 50]. Such trials may offer greater opportunities for LMIC leadership and authorship, and more accurately reflect local health priorities. However, LMIC investigator-led cancer trials remain scarce for various reasons, including lack of funding for investigator-initiated trials and a donors’ focus on research in infectious diseases (e.g. European and Developing Countries Clinical Trials Partnership- for Africa). A recent survey conducted among clinicians in LMICs indicates indeed a demand for increased funding and capacity-building for local (clinical) researchers. But currently, with the United States’ withdrawal from global health initiatives and a decrease in development cooperation elsewhere, there is a risk of further reductions in funding for research on non-communicable diseases in LMICs, as the focus shifts increasingly to health security [51].

One of the most striking, impactful and statistically sound findings of our study is that 78% (21 out of 27 articles) of publications from trials conducted exclusively in MICs had MIC first, middle, and last authors, while 40% (109 out of 275 articles) of publications from trials involving both HIC and MIC sites did not have a single MIC author. This discrepancy suggests that MIC authors are more likely to hold lead and senior roles when trials are geographically centered in MICs, and that the inclusion of HIC sites ‘pushes’ partners apart and reduces MIC authorship. These findings also challenge the assumption that MIC investigators are uninterested in authorship and show that, when given the oppurtunity, they do take key roles.

This has implications for authorship practices and the structure of global partnerships. The comparison between MIC-only and HIC + MIC trials, in particular, suggest the persistence of important power unbalances within certain multinational collaborations.

In our study, we identified a substantial decline in published global clinical trials over the past 10 years. This decline could be related to increased regulatory scrutiny and a greater focus on the ethical implications of trials in lower-resource countries, and/or to a gradual disengagement of commercial sponsors in MICs. It is also possible that trials are now increasingly being registered in other registries, such as the Pan African Clinical Trial Registry (PACTR) https://www.edctp.org/pan-african-clinical-trials-registry/, which are included in the WHO portal, i.e. the International Clinical Trials Registry Platform (ICTRP, https://trialsearch.who.int/Default.aspx*).*

Our study includes some limitations. Firstly, we included in our analysis only industry-sponsored trials (breast, lung and colon cancer) with peer-reviewed publications, thus excluding other forms of scientific dissemination such as pre-prints and conference abstracts. Second, we only retrieved publications for trials registered with ClinicalTrials.gov. This may result in an incomplete database by excluding studies registered in EUDRACT or other WHO-recognised registries. Third, we checked whether a publication had at least one MIC co-author, but we did not examine the specific MIC affiliation of those with authorship. On the other hand, the size of our database -which includes 302 publications from 173 trials- points to a good representativity of the filed over time.

Fourth, our study inclusion criteria might favour HIC authorship, since prior research shows that many trials conducted in LMICs are not industry-sponsored [7]. Moreover, HIC-led trials tend to focus on a limited set of cancer sites, while trials with LMIC-led articles tend to focus on hematologic, gastrointestinal, and breast cancers [6]. However, since we included also breast cancers and colon cancer (which is the most common gastrointestinal cancer), this issue might be a relatively minor concern. Further research should investigate authorship patterns for other cancer types, including some cancers that are highly prevalent in the Global South such as Burkitt lymphoma, and for oncological non-commercial studies. Furthermore, future studies should investigate whether the research conducted in MICs has generated locally-relevant knowledge and has adequately influenced local policies and practices [52].

## Conclusions

Overall, this study found an imbalance in authorship, suggestive of inequities in research partnerships in industry-sponsored breast, lung and colon cancer clinical trials. The sponsors need to work towards greater equity in authorship when collaborating with researchers in LMICs, and oncology researchers and opinion leaders in HICs should actively advocate for more fairness toward their peers in MICs. As this issue seems to have only recently been raised in the specific field of oncology research, more research as well as awareness-raising is needed, to gain further insight into the recognition of authorship and equity in global oncology research partnerships between HICs and LMICs, whether industry- or academia-sponsored.

## Supplementary Information

Below is the link to the electronic supplementary material.

Supplementary Material 1

## Data Availability

Data is provided within the manuscript or supplementary information files. Additional data are available on reasonable request. All data were extracted from public registries & electronic databases, and do not include any personal or medical data. Therefore, ethics review was not needed.
