# Supplementary material for: Assessing patterns of authorship of low- and middle-income countries in global commercial clinical trials in oncology
Source: Global Health. 2025 Nov 22;22:3. doi: 10.1186/s12992-025-01167-8 (PMC12763880; doi:10.1186/s12992-025-01167-8)
Supplement: Supplementary file 1 — Supplementary Material 1 [file 12992_2025_1167_MOESM1_ESM.docx]

**Figure S1** Industry-sponsored clinical trials for breast, lung, and colon cancers conducted in MICs. A.) Industry-sponsored clinical trials for breast, lung, and colon cancers conducted in L-MICs. B.) Industry-sponsored clinical sites for breast, lung, and colon cancers conducted in L-MICs. C.) Industry-sponsored clinical trials for breast, lung, and colon cancers conducted in U-MICs. D.) Industry-sponsored clinical sites for breast, lung, and colon cancers conducted in U-MICs.


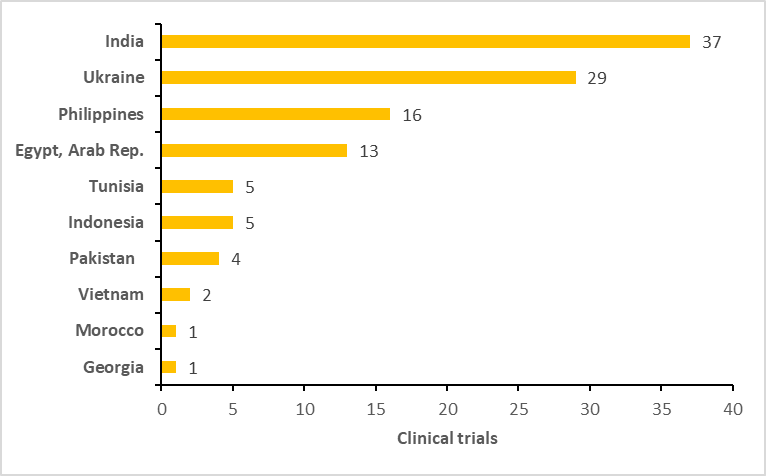

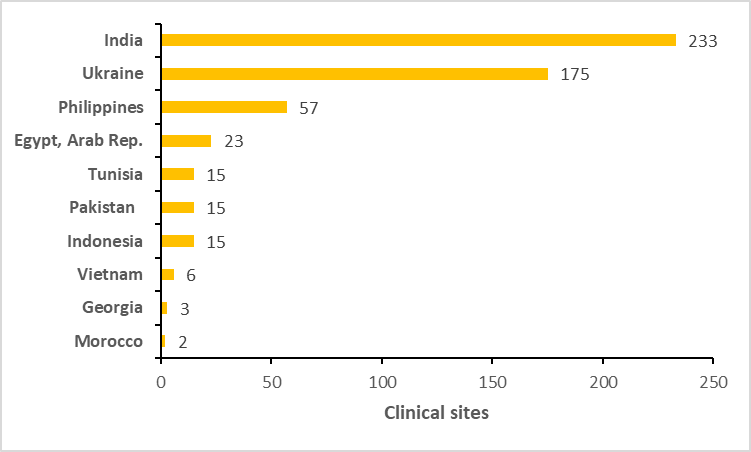


**B**

**A**...

**D**

**C**...


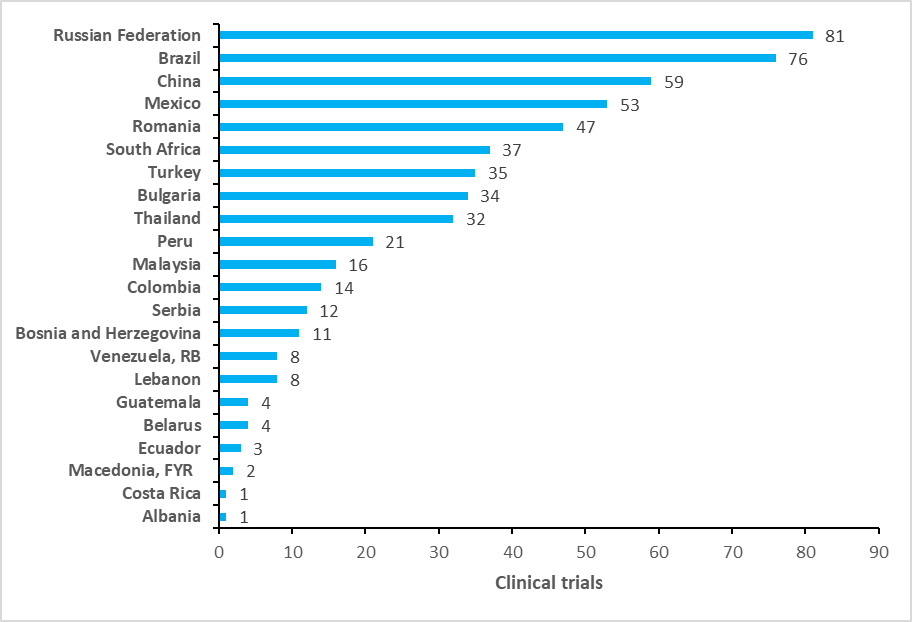

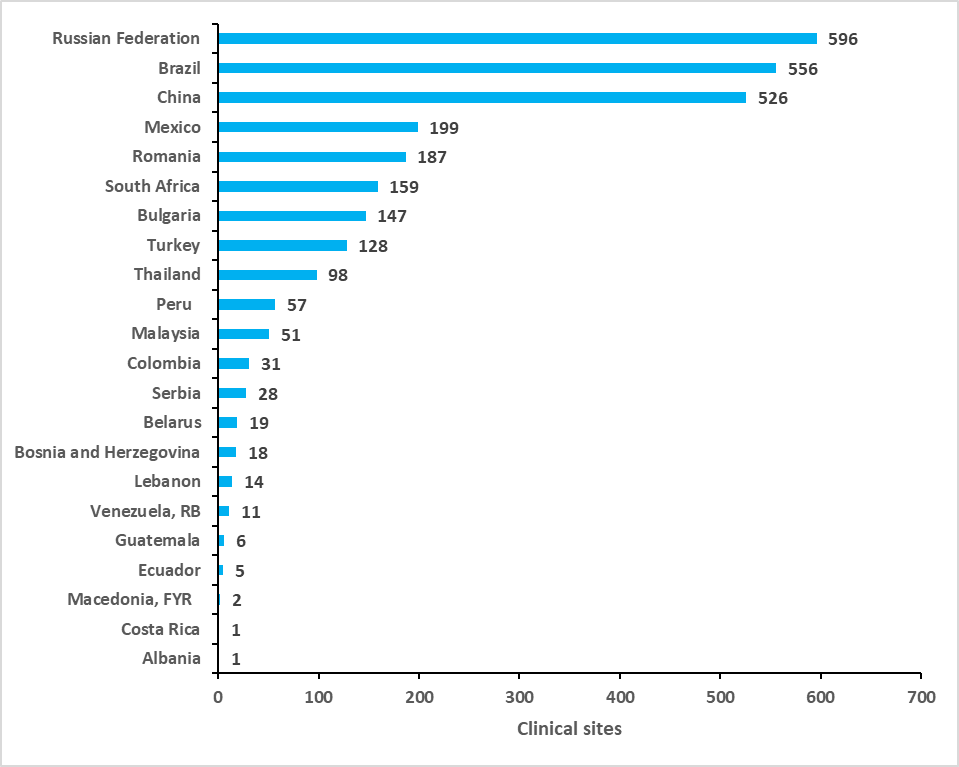


MICs: middle income countries; L-MICs: Lower-middle income countries; U-MICs: Upper-middle income countries
